# Does Information from the Parkinson KinetiGraph™ (PKG) Influence the Neurologist’s Treatment Decisions?—An Observational Study in Routine Clinical Care of People with Parkinson’s Disease

**DOI:** 10.3390/jpm11060519

**Published:** 2021-06-05

**Authors:** Mathias Sundgren, Mattias Andréasson, Per Svenningsson, Rose-Marie Noori, Anders Johansson

**Affiliations:** 1 Department of Neurology, Karolinska University Hospital, 171 64 Stockholm, Sweden; mattias.andreasson@sll.se (M.A.); per.svenningsson@ki.se (P.S.); rose-marie.noori@sll.se (R.-M.N.); anders.c.johansson@sll.se (A.J.); 2 Center for Neurology, Akademiskt Specialistcentrum, Stockholms Läns Sjukvårdsområde (SLSO), 113 65 Stockholm, Sweden

**Keywords:** Parkinson’s disease, objective measurement, PKG, device, treatment

## Abstract

Management of Parkinson’s disease traditionally relies solely on clinical assessment. The PKG objectively measures affected persons’ movements in daily life. The present study evaluated how often PKG data changed treatment decisions in routine clinical care and to what extent the clinical assessment and the PKG interpretation differed. PKG recordings were performed before routine visits. The neurologist first made a clinical assessment without reviewing the PKG. Signs and symptoms were recorded, and a treatment plan was documented. Afterward, the PKG was evaluated. Then, the neurologist decided whether to change the initial treatment plan or not. PKG review resulted in a change in the initial treatment plan in 21 of 66 participants (31.8%). The clinical assessment and the PKG review differed frequently, mainly regarding individual overall presence of motor problems (67%), profile of bradykinesia/wearing off (79%), dyskinesia (35%) and sleep (55%). PKG improved the dialogue with the participant in 88% of cases. PKG and clinical variables were stable when they were repeated after 3–6 months. In conclusion, PKG information changes treatment decisions in nearly a third of people with Parkinson’s disease in routine care. Standard clinical assessment and PKG evaluation are often non-identical. Objective measurements in people living with Parkinson’s disease can add therapeutically relevant information.

## 1. Introduction

Parkinson’s disease (PD) is a common, progressive and serious neurological disorder with a global prevalence of 1–2 per 1000 [1]. The core motor features of PD—bradykinesia, rigidity and tremor—respond to dopaminergic treatment. Adjustments of symptomatic treatments are the focus in the routine clinical care of people with PD (PwPD). Non-motor symptoms such as depression, anxiety, sleep disorders and urinary incontinence may also respond to dopaminergic treatment, underlining the importance of a correct and well-balanced treatment [2]. As the disease progresses, the benefit from each dose of medication becomes shorter, and in many affected people, medication-related excess involuntary movements (dyskinesias) emerge. Motor fluctuations are seen in approximately 40% of PwPD after 4–6 years of treatment and in 70% after >9 years [3,4]. PwPD affected by fluctuations have more disability and poorer quality of life [5].

The signs and symptoms of fluctuations vary considerably and are often under-reported by PwPD and under-recognized by clinicians [6], which makes individualized treatment of people living with PD challenging. Furthermore, patient diaries, clinical rating scales and questionnaires all have well-known limitations in correctly reporting and quantifying symptoms [7]. Although ambulatory objective measurement technologies cannot replace clinical assessment, they have the advantage of passive collection of movement-related data during normal activities of daily living.

The Parkinson’s KinetiGraph™ (PKG) movement recording system is a wrist-worn device carried continuously for 6–10 consecutive days. In addition to motor patterns (bradykinesia, dyskinesia and tremor), the device registers data related to immobility/somnolence, medication adherence and tendency to impulsiveness [8,9]. Target values for different PKG summary scores that can aid clinical evaluations have been proposed [10,11]. The PKG has received regulatory clearance for the use in PwPD in Australia, Europe and the United States.

Repeated clinical visits aided by objective measurements with the PKG improved several outcomes in an Australian cohort of PwPD (Hoehn and Yahr stage: median 2, range 1–3), including the Unified Parkinson’s Disease Rating Scale (UPDRS) part 1–4, quality of life and number of non-motor symptoms [12]. In the same study, PKG was reported to have influenced therapeutic decisions in 61% of cases. Furthermore, a recent blinded longitudinal study showed that PKG-assisted clinical assessments improved the motor score to a significantly higher degree than clinical assessments without PKG information. The PKG-assisted group improved in the Movement Disorder Society-UPDRS (MDS-UPDRS) part 3 mean score from 35.1 to 28.6, while the PKG non-assisted group improved from 35.8 to 33.2 [13].

The overall aim of our study was to evaluate the utility of the PKG when used in routine clinical care of people living with PD. The primary outcome was to evaluate the percentage of treatment decisions that were changed after PKG review. Secondary outcomes were exploratory and included agreement between the clinical assessment and the PKG interpretation, using pre-defined questions regarding motor symptoms, wearing OFF, dyskinesia, sleep, impulse control disorder (ICD) and compliance. Other secondary outcomes were to evaluate whether the PKG improved neurologist-patient dialogue, change in PKG variables, clinical symptoms, health-related QoL, self-rated health state, and non-motor symptoms after follow-up.

Additionally, we compared clinical characteristics and PKG scores in PwPD where treatment plans changed after PKG review to PwPD where it did not.

## 2. Materials and Methods

This prospective, observational cohort study of PwPD attending the Movement Disorder unit at the Karolinska University Hospital and the Akademiskt Specialistcentrum in Stockholm, Sweden was conducted between March 2018 and February 2020. Any consecutive person with PD could be included who could provide informed consent and was judged by the investigator to be able to use the PKG and complete the study. The sample was consecutive from the waiting list but limited by practical aspects. The only exclusion criteria were severe dementia, use of wheelchair more than 50% of the day, and having performed a PKG recording or a visit to the treating neurologist in the last 3 months. The study was approved by the regional ethical board of Stockholm (Regionala Etikprövningsnämnden Stockholm).

At the inclusion visit to the study nurse, approximately 10–20 days before the planned routine visit, a PKG registration was started after informed consent was provided by the participant. In addition, the following information was collected: a short clinical assessment with the Parkinson’s Disease Composite Scale (PDCS) [14], Hoehn and Yahr (H&Y) staging, Non-Motor Symptom Questionnaire (NMSQ) [15], self-assessment of health-related quality of life with Parkinson’s Disease Questionnaire-8 (PDQ-8) [16] and self-rated current health state with the Visual Analogue Scale from the EuroQoL Group (EQ VAS) [17]. NMSQ, PDQ8 and EQ VAS are included as outcome measures in the Swedish Parkinson Register (Available from: https://www.neuroreg.se/, accessed on 30 April 2021) and thus commonly collected in routine care at the participating centers. A higher number in the EQ VAS represents better self-rated health. A higher percentage in the PDQ-8 represents worse self-assessed QoL.

Participants received device instructions and wore the PKG for 6 consecutive days (24 h/day), only temporarily removing it when showering/bathing. The inclusion visit and start of the PKG registration was always on a weekday; thus the registration period also included one or two days of a weekend.

The PKG was uploaded in an electronic case report data base (ActiveReaction Clinical Trials Management System). Five neurologists in the Movement Disorder clinic participated in the study. The group’s average post-residency experience in treating idiopathic PD was 7.3 years (SD 5.7; range 1.5–15.0).

At the study visit, which ranged from 45 to 60 min, the neurologist first completed the clinical evaluation of the routine visit. The PKG data were not available to the neurologist until the end of the planned visit. Before reviewing the PKG results, the neurologist chose from a pre-defined set of alternative descriptions regarding the participant’s clinical condition, covering six items (Table 1). Within each item, it was possible to agree with more than one description, except for ICD and Compliance. After the clinical evaluation, a recommendation regarding the treatment was documented in the electronic case report form. After this, the neurologist reviewed the PKG, which was not technically available before the clinical assessment had been documented. The complete PKG was presented with no external review or comments. The same pre-defined set of alternative descriptions was given again, to be answered based on the inspection of the PKG recording. Then, the physician answered (Yes or No) whether the information obtained from the PKG data changed the first treatment recommendation formulated and documented after the clinical assessment only. During the PKG review, the PwPD usually remained in the office, but there were exceptions when the neurologist asked the person to wait outside for a short period of time.

All participants were included in the primary outcome analysis, irrespective of the recommendation after the standard clinical evaluation. The participant was only given a final treatment recommendation at the end of the visit. Finally, the treating neurologist answered a question if the PKG recording, in his or her opinion, improved the dialogue with the person with PD.

A second PKG recording was performed after 3–6 months, including a new visit to the PD nurse. Secondary outcomes included change in PDCS, NMSQ, PDQ-8, EQ-VAS and PKG variables (Bradykinesia Score/BKS, Dyskinesia Score/DKS and Fluctuations Dyskinesia Score/FDS). Overall change (better/worse) at follow up was rated using the Clinical Global Impressions—Improvement Scale (CGI-I) [18]. A lower score represents improvement and a higher score deterioration. A score of 4 represents no change.

Seventy PwPD were included in the study. Four were excluded after the routine visit due to technical or human errors (e.g., no or wrong PKG uploaded in the electronic case report data base). Clinical characteristics, mean values of clinical measurements and PKG-data of the participants at baseline (*n* = 66) are given in Table 2. PwPD were on average 68.3 years, with a disease duration of 7.9 years. Mean BKS and DKS were 27.9 and 3.5, respectively.

Two participants recorded the first PKG more than 3 weeks before the clinical visit, and five participants completed the follow-up later than 6 months from baseline testing (protocol deviations), but the data were kept in the analysis. Three participants did not complete the follow-up. Of the remaining 63 PwPD, four had missing data in EQ VAS at baseline and/or follow-up.

Statistics: The primary outcome was given as a proportion. Variables were checked for normality (Shapiro–Wilk), and parametric (Paired Samples Test) or non-parametric (Wilcoxon Signed Ranks Test) statistical methods were conducted to compare the means or medians at baseline and follow-up. For each variable in the longitudinal analysis, participants with complete data (at baseline and follow-up) were included. For comparisons of groups, Independent Samples Mann–Whitney U Test and Chi-Square Test were used. Statistical calculations were performed in SPSS 25.0. Significance level was 0.05. No formal a priori sample size calculation was performed. Practical aspects determined the sample size.

## 3. Results

After the clinical assessment alone, the initial treatment plan included a change in treatment for 52 of 66 PwPD. In the remaining 14 participants, the current treatment was planned to be left unchanged. After PKG review, the treatment plan proposed after the clinical assessment alone was changed in 21 of 66 PwPD (31.8%).

The treating neurologist believed that the PKG improved the dialogue with the participant in 58 of the 66 visits (88%).

Clinical characteristics and PKG data were not significantly different between PwPD in which PKG review changed, compared to those where PKG did not change the treatment decision.

PKG inspection differed from clinical assessment (defined as non-identical choices among the pre-defined options) regarding the presence of motor features in 67% of PwPD, characteristics of bradykinesia/wearing off in 79%, dyskinesia in 35%, and sleep in 55%. Almost all participants reported good compliance and no tendency to ICD. For these items, there were few disagreements between the clinical and PKG assessments (for ICD 3% and for compliance 5%). Completely identical answers to the pre-defined options in all six items were only seen in three PwPD.

At follow-up, clinical variables remained stable without significant changes compared to baseline in PDCS, NMSQ, PDQ8 and EQ VAS. BKS improved from 27.9 to 27.4 and DKS changed from 3.5 to 3.2 (not significant) (Table 2). Mean CGIIS was 3.6 at follow-up.

## 4. Discussion

PKG inspection changed the neurologists’ treatment recommendations in 31.8% of the PwPD during routine clinical visits. This indicates that PKG data often provide clinically and therapeutically important information in routine care of PwPD. The study did not grade the magnitude of change, which ranged from minor (e.g., slightly adjusting the timing of a single dose) to more extensive changes (e.g., adding or stopping a drug). There were no changes in clinical or PKG parameters at follow-up, but the study was not powered for this.

Our results are comparable to reports from previous studies. A recent study reported that PKG data led to a final adjustment of the medication in 36 of 112 PwPD (32%) during routine care [19]. In another study on a similar study population as ours and with a similar objective, the clinical decision changed in 24 of 70 British PwPD (34%) based on the results of PKG recordings [20].

Higher proportions regarding the influence of PKG on therapeutic decisions have been reported. Farzanehfar et al. reported that PKG data influenced therapeutic decisions in 61% of the participants in a cohort of Australian PwPD seen in routine care [12]. In an American study, PKG-data influenced treatment plans in 79% of studied PwPD, but the method of determining this was not detailed [21]. An advantage of our study was that the initial treatment plan, before the PKG was reviewed, had to be documented to get access to the PKG. This procedure made distinguishing “change” from “no change” of treatment decisions after the PKG review simple, and it minimized potential bias. Thus, our results add to the growing body of evidence that objective measurements with the PKG influence clinical practice.

There was frequently a difference in the detailed interpretation, especially regarding the profile of bradykinesia/wearing OFF and the presence of different motor symptoms. This finding is in line with a previous report that PKG yielded important new information primarily regarding OFF time [19]. A recent blinded study compared PKG-assisted clinical evaluations with clinical assessments where PKG data were not revealed to the neurologist. In the group with available PKG data, motor function (MDS-UPDRS part 3) improved significantly more than in the control group. The difference was largest for PwPD who had a high bradykinesia score at baseline, who thus benefited most from PKG evaluations [13].

We only analyzed the answers in the pre-defined set of options for each item as either identical or not identical, thus not grading the magnitude of disagreements. There are many possible natural explanations for these different interpretations. A clinical evaluation usually covers a longer time period than the 6-day PKG recording, or the PKG period may not have been felt to be representative by the person living with PD or the treating neurologist. Some features in the PKG may represent artifacts such as physical exercise (increasing DKS) or excessive daytime somnolence (increasing BKS). One study reported that 10 of 103 PKG recordings were unrepresentative due to such artifacts [12]. Furthermore, it is important to acknowledge that the PKG system has limitations due to its inability to detect some of the most troublesome symptoms. In this study, a few options (e.g., “freezing” in item Motor symptoms and “night-time sleep issues” in item Sleep) are difficult or impossible to detect in the PKG. Regarding dyskinesias, we cannot exclude that the presence of axial dyskinesias might have contributed to disagreements between the clinical and wrist-worn PKG assessments.

Degree of experience in using and interpreting PKG recordings may also influence results. Several descriptive options given to the neurologist, from which more than one could be chosen, increase the chances of results being non-identical. Furthermore, in many cases, the disagreements between the clinical and the PKG assessments were not large or clinically meaningful. For example, a disagreement could be that the clinical assessment resulted in the selection of two descriptions for bradykinesia and the inspection of the PKG for the same person resulted in selecting the same two options plus one more.

Despite these possible explanations, it is reasonable to argue that PKG data often provide clinical details not detected during routine clinical assessments. This study shows that in a substantial proportion, this additional data led the neurologist to change, refine or adjust the treatment plan. The present study does not support the possibility that PKG provides substantially different information about ICD or compliance compared to asking the PwPD about these aspects during the clinical visit. It is possible that the reminder signal, inherent to the device, might have increased compliance and thus clouded the results of this specific outcome. Similar results were reported in a previous PKG study. Of 85 visits, no cases of ICD or non-adherence to medication were observed in the PKG [21].

The PKG allows for a graphical description of the symptom profile that can be shared and explained to the person living with PD. In the present study, the participating neurologists considered that the PKG improved the dialogue with the participant in nearly 9 of 10 visits. Our results also indicate that treating neurologists do not overemphasize the PKG data, because in many cases, a disagreement between the clinical and the PKG interpretation did not lead to a change in the initial treatment decision. A previous study reported that PKG provided additional information resulting in a change in the care for the majority, but for 22% of the PwPD, the PKG data did not alter treatment [19].

Clinical and PKG data for those in which PKG review led to a change in treatment were not different from those in which it did not, indicating difficulties in predicting which PwPD will benefit from PKG evaluations.

BKS < 25 has been suggested as an appropriate target for acceptable bradykinesia in PwPD [10,11]. The mean BKS of 27.9 in our group of participants is above this proposed target level, and it is also higher than the average BKS reported in a large analysis of previous PKG recordings in Sweden (25.0) [22]. This suggests that the present study included PwPD that on average were more poorly controlled. Nevertheless, a large proportion of PwPD globally seem to be undertreated, as judged by BKS scores. Analysis of a large PKG data base showed that, depending on the country, 46 to 61% of people living with PD had a BKS > 25 [22].

No significant changes in clinical scales or PKG data were seen at follow up. PwPD with repeated PKG-recordings tend to show improved BKS and DKS data over time [22]. In the present study, BKS improved from 27.9 to 27.4 and DKS from 3.5 to 3.2, but these changes were not statistically significant. A mean CGIIS of 3.6 reflects a slight overall improvement.

The present study did not include defined PKG targets because target values had not been proposed when this study was planned (2017). It is possible that the results of this study would have been different with such targets.

We believe the PKG is a valuable tool for better understanding the present clinical condition of PwPD, but it needs to be interpreted together with a clinical assessment that includes a detailed history and a clinical exam. Only relying on PKG data for therapeutic decisions is not recommended. A previous PKG study reported that 17% of PwPD in routine care who had PKG data indicating they were clinically uncontrolled were considered unsuitable for increased treatment because of the risk of side effects [12].

A few limitations in this study should be noted. The number of participants was too low to detect significant changes at follow-up. PKG is validated for people aged 46–83, and the present study included two persons outside this interval (37 and 86 years, respectively). PKG data on immobility and tremor was not included in the separate analysis of PKG data but was available to the treating neurologist when inspecting the PKG. The study was not blinded, and the treating neurologists may unintentionally have performed the clinical evaluation more thoroughly than normal. The way an improved dialogue between neurologists and PwPD was determined was not checked for face validity.

## 5. Conclusions

Objective and passive movement analysis using PKG data changes treatment decisions in almost a third of PwPD in routine clinical care. PKG provides supplementary information to the standard clinical assessment because these are often non-identical, specifically regarding the presence of different motor symptoms, presence and profile of wearing off and dyskinesia, and presence and profile of sleep disturbances. PKG indicators for ICD and compliance are less likely to provide information that is different from the clinical evaluation alone. Objective measurement in people living with PD holds promise for better clinical evaluations. Future randomized control group studies with clearly defined outcome measures are needed to further evaluate if objective measurement improves treatment outcomes in people with PD.

## Figures and Tables

**Table 1:** Pre-defined set of options for describing the participant’s clinical state. The treating neurologist chose one or several options for each item (except for ICD and Compliance, where only one answer could be chosen). The set of options was given twice, first based on the clinical assessment, and again based on the PKG findings.

**Motor Symptoms Present**
■ Bradykinesia
■ Dyskinesia
■ Freezing
■ Tremor
■ Other (the neurologist asked to specify)
**Bradykinesia and Wearing OFF**
■ No bradykinesia or wearing OFF
■ Bradykinesia (or OFF-periods) dominates over significant parts of the waking time
■ Bradykinesia (or wearing OFF) occurs after one or more doses of dopaminergic drug
■ Unpredictable periods of bradykinesia (or OFF periods), or delayed/missing effect of dopaminergic drug
■ Bradykinesia (or OFF-periods) in the morning before the first dose of dopaminergic medicine
■ Bradykinesia or wearing OFF occurs but unclear in what relation to dopaminergic drug treatment
**Dyskinesia**
■ No dyskinesia
■ Dyskinesia dominates during significant periods of the waking time
■ Dyskinesia appears in a predictable pattern after one or more doses of dopaminergic medication (peak dose dyskinesia)
■ Dyskinesia appears but in an unpredictable pattern, e.g., during bradykinesia/OFF or biphasic pattern
■ Dyskinesia appears to occur but unclear in what relation to dopaminergic drug treatment
**Impulse Control Disorder**
■ ICD present
■ ICD not present
**Sleep**
■ No sleep related issues
■ Daytime somnolence
■ Dose related fatigue
■ Night time sleep issues
**Compliance**
■ Good compliance
■ Poor compliance

**Table 2:** Participant characteristics, clinical variables and PKG-data at baseline and follow-up. Results are given in means (min–max). Changes in clinical variables and PKG data were not significant.

	Baseline (*n* = 66)	Follow-Up (*n* = 63)	*p*-Value
Sex (male), %	51.5		
Age, years	68.3 (37–86)		
PD-duration, years	7.9 (0.9–24.3)		
H&Y	2.1 (1–4)		
PDCS	18.5 (1–37)	18.8 (1–40)	n.s.
NMSQ	9.0 (0–18)	8.8 (1–17)	n.s.
EQ VAS	66.0 (20–98) (*n* = 64)	66.7 (25–98) (*n* = 60)	n.s.
PDQ8	22.7 (0.0–46.9)	21.5 (0.0–53.1)	n.s.
BKS	27.9 (12.1–43.8)	27.4 (13.4–42.0)	n.s.
DKS	3.5 (0.1–37.5)	3.2 (0.1–17.2)	n.s.
FDS	8.5 (3.0–22.6)	8.6 (3.8–17.3)	n.s.

## Data Availability

The anonymized datasets used and/or analyzed during the current study are available from the corresponding author on reasonable request.
